# Live Cell Microscopy of Murine Polyomavirus Subnuclear Replication Centers

**DOI:** 10.3390/v12101123

**Published:** 2020-10-02

**Authors:** Douglas K. Peters, Kimberly D. Erickson, Robert L. Garcea

**Affiliations:** 1 BioFrontiers Institute, University of Colorado Boulder, Boulder, CO 80309, USA; Douglas.Peters@colorado.edu (D.K.P.); Ericksk@colorado.edu (K.D.E.); 2 Department of Molecular, Cellular, and Developmental Biology, University of Colorado Boulder, Boulder, CO 80309, USA

**Keywords:** murine polyomavirus, viral replication centers, live cell microscopy, replication protein A, DNA damage response, T antigen

## Abstract

During polyomavirus (PyV) infection, host proteins localize to subnuclear domains, termed viral replication centers (VRCs), to mediate viral genome replication. Although the protein composition and spatial organization of VRCs have been described using high-resolution immunofluorescence microscopy, little is known about the temporal dynamics of VRC formation over the course of infection. We used live cell fluorescence microscopy to analyze VRC formation during murine PyV (MuPyV) infection of a mouse fibroblast cell line that constitutively expresses a GFP-tagged replication protein A complex subunit (GFP-RPA32). The RPA complex forms a heterotrimer (RPA70/32/14) that regulates cellular DNA replication and repair and is a known VRC component. We validated previous observations that GFP-RPA32 relocalized to sites of cellular DNA damage in uninfected cells and to VRCs in MuPyV-infected cells. We then used GFP-RPA32 as a marker of VRC formation and expansion during live cell microscopy of infected cells. VRC formation occurred at variable times post-infection, but the rate of VRC expansion was similar between cells. Additionally, we found that the early viral protein, small TAg (ST), was required for VRC expansion but not VRC formation, consistent with the role of ST in promoting efficient vDNA replication. These results demonstrate the dynamic nature of VRCs over the course of infection and establish an approach for analyzing viral replication in live cells.

## 1. Introduction

Viruses are obligate intracellular pathogens that have developed diverse mechanisms to utilize host cell factors to permit and enhance infection. Not only must these cellular factors be successfully co-opted, but their activities also must be regulated spatially and temporally to ensure productive and efficient infection [1]. DNA viruses require many of the same components used by cells to replicate their genomes and thus have evolved strategies to hijack cellular DNA replication and repair pathways during infection. DNA viruses, including polyomaviruses (PyVs), adenoviruses, papillomaviruses, and herpesviruses, form specialized nuclear compartments during infection that spatially organize replication, transcription, and viral assembly [2,3,4]. We investigated the spatiotemporal dynamics of compartments formed during murine PyV (MuPyV) infection, termed viral replication centers (VRCs) [5,6,7].

The MuPyV genome is ~5.3 kbp of double-stranded DNA, organized as a circularized minichromosome associated with 24 host nucleosomes [8,9]. The MuPyV genome encodes six proteins, three tumor antigens proteins (TAgs), and three capsid proteins (VP1-3). The TAgs are expressed early in infection, and are termed large TAg (LT), middle TAg (MT), and small TAg (ST) based on their relative sizes. These multifunctional proteins prime the cell for viral replication by interacting with critical cellular proteins and forcing the cell into a prolonged S-phase [10,11,12]. LT is enriched at VRCs and acts as helicase for viral DNA (vDNA) replication [13,14]. MT mimics a membrane-bound activated protein tyrosine kinase receptor, modulating cell signaling pathways to enhance virus replication [15,16,17]. ST is a soluble protein that binds protein phosphatase 2A (PP2A), thus affecting the phosphorylation and activation state of cellular proteins controlling the cell cycle and DNA replication and repair [12,18,19]. Mutant viruses lacking MT and/or ST have been used to analyze how each of these protein interactions influence virus replication [6,20,21,22].

VRCs are minimally defined as nuclear domains where LT and vDNA colocalize, although many cellular proteins are associated with VRCs [5,6,7]. For example, immunofluorescence microscopy of fixed, infected cells has shown that proteins involved with cellular DNA replication and repair are enriched at VRCs [5,6,7,23]. As with other DNA viruses, PyVs engage with the cellular DNA damage response (DDR) during infection. In particular, the activity of double-strand break repair and homologous recombination repair proteins are critical for PyV infection, possibly to resolve the Cairns intermediate that results from bidirectional replication of the viral genome [4,6,24,25,26]. We previously used super-resolution fluorescence microscopy of fixed cells to define vDNA replication- and repair-associated subdomains within MuPyV replication centers that are spatially and functionally distinct [7]. LT localized to the replication-associated subdomain, whereas DDR signaling proteins, such as phosphorylated ataxia-telangiectasia mutated kinase (pATM^S1981^), localized to the repair-associated subdomain. The host protein complex, replication protein A (RPA), associated with both subdomains, reflecting the roles of RPA in both DNA replication and repair processes [27,28]. RPA is highly conserved across species and is integral for binding and stabilizing single-stranded DNA (ssDNA) during DNA replication and repair. In mammalian cells, RPA exists as a heterotrimer complex composed of RPA70, RPA32, and RPA14 subunits. SV40 LT binds the RPA70 and RPA32 subunits during vDNA replication, and co-immunoprecipitation of transiently expressed RPA subunits indicates that MuPyV LT may also interact with RPA70 and RPA32 [29,30,31,32]. Together, these results suggest that LT binding to RPA contributes to efficient RPA loading onto ssDNA that emerges behind the LT helicase.

Little is known concerning the formation and maturation of VRCs throughout the course of infection. MuPyV replication centers appear to expand from small foci into large tracts as vDNA replication progresses, but this expansion has previously only been studied in chemically fixed cells, hampering any temporal analysis of VRC dynamics. We therefore investigated VRC dynamics in live cells by generating a mouse fibroblast cell line that stably expresses human RPA32 fused to a green fluorescence protein (GFP-RPA32). We assessed the relocalization of GFP-RPA32 to sites of cellular DNA damage, as well as the colocalization of GFP-RPA32 with known VRC components in fixed cells. Live cell microscopy of the GFP-RPA32 signal was used to visualize VRCs over the course of infection, and an analysis protocol was developed to quantify changes in GFP-RPA32 localization on a single cell basis. Using live cell microscopy, we examined how viral multiplicity of infection (MOI) affects VRC dynamics and analyzed how the MT and ST proteins contribute to the appearance and expansion of VRCs.

## 2. Materials and Methods

### 2.1. Cell Lines and Culture

C57BL/6 mouse embryonic fibroblasts (MEFs) were obtained from ATCC (Manassas, VA, USA, Cat. #SCRC-1008) and used to generate stable cell lines. All cell lines were grown in Dulbecco’s Modified Eagle’s Medium (DMEM; Sigma-Aldrich, St. Louis, MO, USA, Cat. #D6429) supplemented with 10% fetal bovine serum (FBS; Sigma-Aldrich, St. Louis, MO, USA, Cat. #F0926), 1× antibiotic-antimycotic (A-A; Gibco, Carlsbad, CA, USA, Cat. #15240062), and 55 μM β-mercaptoethanol (βME) at 37 °C with 5% CO_2_. Hydroxyurea (HU; Sigma-Aldrich, St. Louis, MO, USA, Cat. #H8627) was dissolved in DMEM/10% FBS/A-A/βME to a concentration of 5 mM for all treatments.

### 2.2. Stable Cell Line Generation

Cell lines stably expressing GFP-RPA32 or GFP-tagged nuclear localization sequence (GFP-NLS) were generated using the PiggyBac Transposon System (System Biosciences). C57BL/6 MEFs were co-transfected with 1 μg of the transposase vector and 1 μg of the ITR-expression cassette-ITR vector (see Section 2.3 Plasmids for description). Lipofectamine 2000 (Invitrogen, Carlsbad, CA, USA, Cat. #11668019) was used for all transfections, as per the manufacturer’s protocol. Cells were selected with 50 μg/mL Hygromycin B (Thermo Fisher Scientific, Waltham, MA, USA, Cat. #10687010) starting at two days post-transfection and maintained under antibiotic selection until established (approximately 1–2 weeks).

### 2.3. Plasmids

The GFP-RPA32 plasmid (gift of Dr. Marc Wold, University of Iowa, Iowa City, IA, USA) was restriction-digested with NheI and BamHI and ligated into corresponding sites in pLP28, a modified PiggyBac ITR-expression cassette-ITR expression vector (gift of Dr. Amy Palmer, University of Colorado Boulder, Boulder, CO, USA), using T4 DNA ligase (NEB, Ipswich, MA, USA, Cat. #M0202T) as per the manufacturer’s protocol. The GFP-NLS plasmid (gift of Dr. Amy Palmer, University of Colorado Boulder, Boulder, CO, USA) was restriction-digested with NheI and NotI and ligated into pLP28 as described above. The ligation products were each transformed into DH5α *Escherichia coli* and selected on ampicillin agar plates. Positive clones were screened by restriction-digestion with NheI and BamHI and the plasmids were confirmed with DNA sequencing.

### 2.4. Viruses and Infections

The MuPyV strain NG59RA was used for all wild-type (WT) virus infections [33]. Virus strains NG18 and NG59 have mutations that functionally eliminate MT and ST expression [20,21,34,35]. Virus 808A has a mutation in the MT splice acceptor that prevents the expression of MT (expression of LT and ST are unaffected) [21,22]. Infections were carried out as described previously [7]. Briefly, cells were grown to 40% confluency and then cultured overnight in DMEM/A-A/βME without serum. Virus was diluted in an adsorption buffer (Hanks Balanced Salt Solution (HBSS)/10 mM HEPES, pH 5.6/0.5% bovine calf serum (BCS)) and added to cells for 1–2 h at 37 °C and 5% CO_2_, after which the viral supernatant was removed and replaced with post-infection media (DMEM/1% FBS/A-A/βME) for the remainder of the experiment. Unless stated otherwise, cells were infected at a MOI that yields 30% infection efficiency.

### 2.5. Microscopy

#### 2.5.1. Laser Scanning Confocal Microscopy

For laser scanning confocal microscopy (LSCM) of fixed samples, cells were cultured on acid-etched glass coverslips (12 mm, No. 1.5) and infected as described above. Cells were pre-extracted and fixed as described previously [6,7]. After fixation, cells were blocked overnight at 4 °C in 10% BCS/PBS (block solution), incubated with primary antibody diluted in block solution at 37 °C for 1 h, and then rinsed three times with 4 °C block solution. Cells were then incubated for 1 h at 37 °C with Alexa Fluor-conjugated secondary antibodies diluted in block solution, rinsed three times with PBS, and mounted onto slides with ProLong Gold Antifade Mountant with DAPI (Invitrogen, Carlsbad, CA, USA, Cat. #P36931). Samples were cured at room temperature (RT) for at least 24 h before imaging. LSCM images were acquired on a Nikon A1R microscope, using a 1.45 NA 100× oil objective, 405/488/561/640 laser lines, and photomultiplier tube (PMT) detectors.

For LSCM of live cells, GFP-RPA32-expressing cells were grown on glass imaging dishes (MatTek Life Sciences, Ashland, MA, USA, Cat. #P35G-1.5-20-C) until 50% confluent. Thirty minutes prior to imaging, the growth media were replaced with Fluorobrite DMEM (Gibco, Cat. #A18967) containing 10 µg/mL Hoechst 33342 dye (Invitrogen, Carlsbad, CA, USA, Cat. #H3570). Cells were maintained in an environmental chamber at 37 °C, 70% humidity, and 5% CO_2_ throughout the experiment and imaged on a Nikon A1R LSCM using a 1.3 NA 40× oil objective. To induce DNA damage, a region of interest (ROI) within the nucleus was defined using the microscope software (Nikon Elements) and damage was induced within the ROI using the 405 nm laser operating at 50% power for 1 min. Cells were imaged prior to UV irradiation, then at 30 s intervals after irradiation for a maximum of 20 min. Images were recorded at a single z-plane using PMT detectors.

#### 2.5.2. High-Throughput Widefield Microscopy

For infectibility experiments, cells were grown on 96-well imaging dishes (Corning Costar, Cat. #3904) and infected as described above. At 28 h post-infection (HPI), cells were fixed in 4% paraformaldehyde (Electron Microscopy Sciences, Hatfield, PA, USA, Cat. #15714), diluted in PBS for 15 min at room temperature (RT), permeabilized in 0.5% Triton X-100 (Acros Organics, Geel, Belgium, Cat. #215682500) for 5 min at RT, and immunolabeled as described above for LSCM samples. This protocol does not remove soluble nuclear proteins prior to fixation and immunolabeling, unlike the pre-extraction protocol described above for the LSCM images. Infectibility experiments were imaged on a Molecular Devices ImageXpress MicroXL Ultra Widefield microscope. A 0.45 NA 10× air objective was used, and images were recorded using a PCO sCMOS camera with 6.5 μm pixels. A single z-position was imaged at each site.

#### 2.5.3. Spinning Disc Confocal Microscopy

For each time course experiment, cells were grown on glass-bottom 96-well imaging plates (MatTek Life Sciences, Ashland, MA, USA, Cat #PBK96G-1.5-5-F) and infected as described above. All live cell microscopy images were acquired on a Nikon TiE spinning disc confocal (SDC) microscope with a Yokogawa CSU-X1 environmental chamber, unless otherwise noted. Cells were maintained in an environmental chamber at 37 °C, 70% humidity, and 5% CO_2_ throughout the experiment. A 0.95 NA 40× air objective was used, and images were recorded using an Andor 888 Ultra EMCCD (13 μm pixels). A single z-position was imaged throughout each time course experiment.

Immunofluorescence images of fixed samples (post-live cell imaging) were acquired on the same microscope. Cells were removed from the microscope and immediately fixed, permeabilized, and immunolabeled as described above for widefield microscopy. Upon re-imaging, the same fields of cells were rediscovered using the X/Y coordinates recorded for the live cell portion of the experiment.

### 2.6. Immunofluorescence Antibodies

The primary antibodies used for immuno-labeling were: rat anti-TAg (E1, 1:2000, gift from Tom Benjamin); rat anti-RPA32 (4E4, 1:10, gift from Heinz-Peter Nasheuer) [36]; mouse anti-pATM^S1981^ (clone 10H11.E12, 1:250, Millipore Sigma, Burlington, MA, USA); rabbit anti-γH2AX (ab11174, 1:1000, Abcam, Cambridge, UK); rabbit anti-VP1 (I58, 1:5000) [37]. Primary antibodies were detected using secondary antibodies conjugated with Alexa Fluor 488, Alexa Fluor 546, or Alexa Fluor 647 (Invitrogen), and diluted to 1:2000. All primary and secondary antibodies were diluted in blocking solution.

### 2.7. Image Processing

Image processing was performed using ImageJ analysis software (NIH). For representative images, single z-planes are shown. Brightness and contrast were adjusted when necessary to display the full dynamic range of each image. When multiple images of the same cell were displayed (i.e., at different time points), brightness and contrast were kept consistent to enable comparisons of relative signal intensities.

### 2.8. Image Analysis

#### 2.8.1. Line Scan Analysis

Line scan analysis was carried out using ImageJ to illustrate the spatial relationships of fluorescent signals, as described in [7]. Briefly, a line was drawn through an area of interest in an image and the signal intensity of each pixel along that line was recorded in each fluorescent channel using the Plot Profile tool. The values of each signal intensity were then normalized to the minimum and maximum values of each channel to yield a common value scale from 0–1.

#### 2.8.2. Infectibility Assay

Infectibility assay image analysis was performed using a custom MatLab^®^ script. Briefly, individual nuclei were identified in each image based on Hoechst signal. The mean nuclear signal intensity of LT and VP1 signals was calculated for each cell, and a threshold was set to determine which cells were infected. The numbers of infected and total cells were summed across five sites in each well and then the proportion of infected cells was calculated and reported as a single replicate. Each condition was repeated in quadruplicate.

#### 2.8.3. Spatiotemporal Analysis of Live Cell Microscopy

Live cell imaging data were analyzed using a combination of ImageJ and MatLab. The analysis rationale is outlined in the text and in Section 3.3 and Figure S2. Cells were excluded from analysis if they left the field of view or went out of focus during the time course. Using ImageJ, individual nuclei were manually tracked and cropped from several frames of raw live cell imaging data. Unless noted otherwise, one frame was analyzed per hour of imaging. A custom MatLab script was used to segment nuclear pixels from the image background in each frame and for each cell. Individual time points were excluded from analysis if nuclear segmentation was inaccurate, which typically only affected images of cells approaching the lytic end of infection. An intensity threshold was used to distinguish “diffuse” and “focal/VRC” pixels within the nucleus. A floating intensity threshold was calculated for each nucleus in each frame to accommodate differences in GFP-RPA32 expression, nuclear volume, and infection timing between cells. For each nucleus in each frame, pixels that were at least 1.5-times brighter than the mean intensity were designated “VRC pixels”. This threshold was used because it minimized the detection of small fluctuations in GFP-RPA32 localization while still remaining sensitive to the formation and expansion of VRCs. The floating threshold was relatively stable between time points within the same cell because the mean pixel intensity did not change significantly throughout the time course (Figure S2C).

The integrated signal intensity of all VRC-associated pixels was divided by the integrated signal intensity of the entire nucleus to yield the “percentage of GFP-RPA32 signal in VRCs”. The number of VRC-associated pixels was divided by the total nuclear area to yield the “percentage of nuclear area in VRCs”. The signal intensities of all VRC-associated pixels were averaged and then divided by the mean intensity of the nucleus to yield the “mean VRC pixel intensity”.

For temporal alignment in Section 3.4 and Section 3.5, values for each cell (the cell “trace”) were offset in time and aligned to the frame at which they were nearest a threshold of 5% of Signal in VRCs. When multiple different metrics were aligned (Section 3.5), the offset value of each cell trace was kept at the same value (i.e., it was based on the 5% of Signal in VRCs threshold).For quantification of VRC number (Section 3.4 and Section 3.5), GFP-RPA32 foci were manually quantified at the threshold time point (i.e., the frame in which each cell passed the 5% of Signal in VRCs threshold).

### 2.9. Statistical Analysis

All error bars represent standard deviation. Statistical analyses were carried out in GraphPad Prism software (version 8.4.3). *p* values < 0.001 are indicated with ***, and *p* values > 0.05 are indicated with “ns”.

## 3. Results

### 3.1. GFP-RPA32 Relocalizes to Cellular DNA Damage Foci

**Figure 1:**
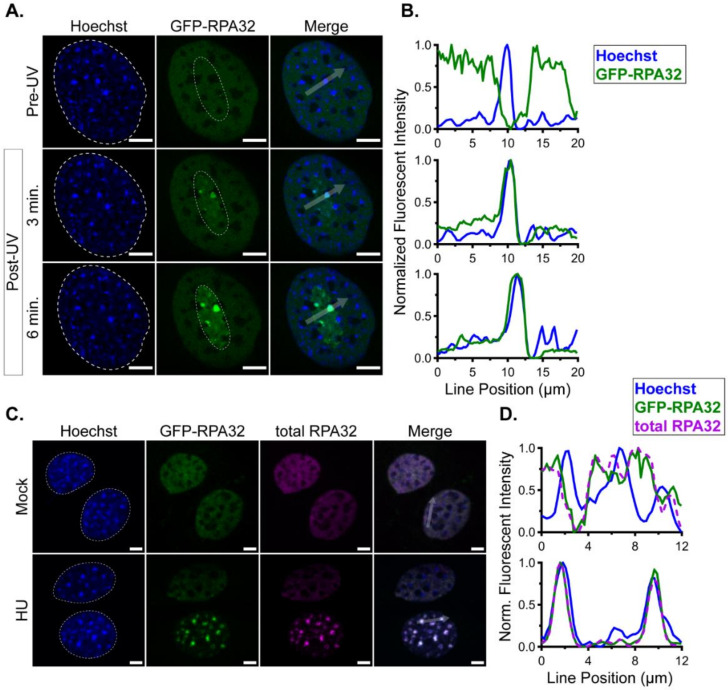
GFP-RPA32 relocalizes to cellular DNA damage foci. Mouse cell lines stably expressing GFP-RPA32 were imaged before and after induction of cellular DNA damage by UV irradiation (405 nm laser) or 5 mM hydroxyurea (HU). (**A**) Hoechst (blue, left column), GFP-RPA32 (green, middle column), and merge (right column) images are shown of a representative cell at multiple time points: pre-UV irradiation (top row), 3 min post-UV (middle row), and 6 min post-UV (bottom row). The nuclear area is denoted by a dotted white line in each Hoechst image, and the UV-irradiated region is denoted by a thin dotted white line in each GFP-RPA32 image. (**B**) Line scan analysis of Hoechst (blue) and GFP-RPA32 (green) signals along the white arrow in each merge image in (**A**). Fluorescence intensities were analyzed for each fluorescent channel and normalized to min and max values within each channel. (**C**) Hoechst (blue), GFP-RPA32 (green), total RPA32 (magenta), and merge images are shown of representative cells 1 h after treatment with hydroxyurea (HU)-free media (Mock, top row) or media containing 5 mM HU (bottom row). Nuclear borders are denoted in each Hoechst image by dotted white lines. (**D**) Line scan analysis of Hoechst (blue), GFP-RPA32 (green), and total RPA32 (magenta) signals along the white arrows in each merge image in (**C**). Fluorescence intensities were analyzed as in (**B**). Scale bar = 5 μm.

To visualize and measure VRCs in live cells, we generated a mouse fibroblast cell line that stably expressed a GFP-tagged human RPA32 (GFP-RPA32). To verify the localization and activity of this GFP-RPA32 protein in mouse cells, GFP-RPA32-expressing cells were imaged using a laser scanning confocal microscope (LSCM) before and after induction of cellular DNA damage by UV irradiation. Hoechst dye was used to label nuclei and to sensitize DNA to UV-induced damage. In unirradiated cells, GFP-RPA32 exhibited a diffuse, nuclear localization while excluded from heterochromatin and nucleoli (Figure 1A, top row) [7,38,39]. Following UV irradiation of a defined region of interest (ROI), GFP-RPA32 became progressively enriched within the irradiated region (Figure 1A, middle and bottom rows). Line scan analysis confirmed the relocalization of GFP-RPA32 after irradiation, particularly to heterochromatin (Figure 1B and Figure S1A). Additional analysis revealed a large dynamic range of signal intensities (approximately 16-fold) between different regions of irradiated nuclei, further indicating shifts in GFP-RPA32 localization after UV irradiation (Figure S1A). As a control, cells constitutively expressing a GFP-tagged nuclear localization sequence (GFP-NLS) were irradiated, and, as predicted, GFP-NLS did not relocalize after UV irradiation (Figure S1B,C). These results confirmed that GFP-RPA32 specifically relocalizes to DNA damage sites in response to UV irradiation.

GFP-RPA32- and GFP-NLS-expressing cells were treated with hydroxyurea (HU), fixed, and total RPA32 was immunolocalized using an antibody that recognized both mouse and human RPA32 proteins. HU inhibits ribonucleotide reductase, thereby rapidly depleting the pools of deoxynucleotide triphosphates (dNTPs) available for DNA synthesis. In cells that are in S-phase, the depletion of dNTPs leads to stalled replication forks, which exhibit extended RPA-bound ssDNA and DDR signaling by a different pathway than UV irradiation [40,41,42]. GFP-RPA32-expressing cells treated with HU exhibited total RPA32 foci that were stable over time and colocalized with GFP-RPA32 signals, whereas mock-treated cells had no such foci (Figure 1C,D). GFP-NLS-expressing cells treated with HU also displayed total RPA32 foci, but GFP-NLS signal remained diffuse (Figure S1D,E). Taken together, these data show that the human GFP-RPA32 localizes to sites of DNA damage in mouse cells.

### 3.2. GFP-RPA32 Localizes to VRCs in MuPyV-Infected Cells

**Figure 2:**
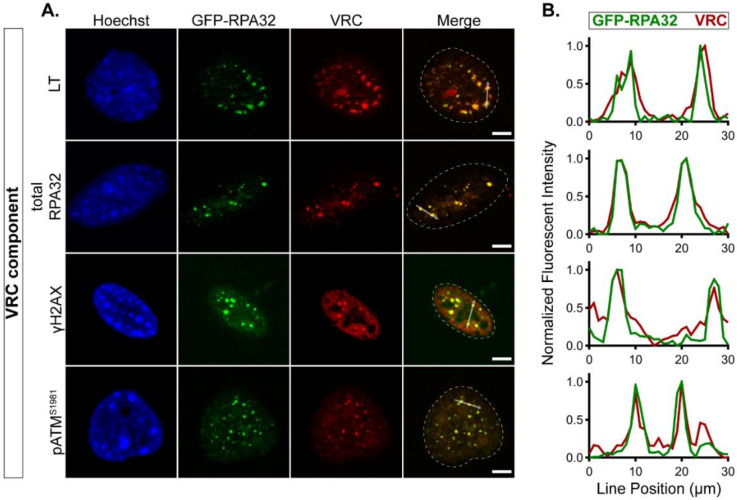
GFP-RPA32 relocalizes to viral replication centers (VRCs) in murine polyomavirus (MuPyV)-infected cells. GFP-RPA32-expressing cells were infected for 28 h and processed for immunofluorescence microscopy. (**A**) One z-position of a representative cell is shown for each immunolabeled VRC component designated on the left: large tumor antigen protein (LT), total RPA32 (endogenous and GFP-RPA32), γH2AX, and pATM^S1981^. Channels: Hoechst = blue, GFP-RPA32 = green, VRC components = red. The nuclear border is denoted by a dotted white line in each merge image. Scale bar = 5 μm. (**B**) Line scan analysis of GFP-RPA32 (green) and VRC component (red) signals along the white arrow in each merge image in (**A**). Fluorescence intensities were analyzed and normalized as in Figure 1.

To determine if GFP-RPA32- and GFP-NLS-expressing cells were permissive for MuPyV infection, we assessed the expression of the viral proteins LT and VP1 across a range of MOIs and found that the cell lines were comparably infectible (Figure S1F). To confirm that GFP-RPA32 relocalized to VRCs, we immunolabeled known VRC components in the GFP-RPA32-expressing cells (Figure 2A,B) [5,6,7,23]. We found that GFP-RPA32 colocalized with both LT and total RPA32 (endogenous and GFP-RPA32), as well as γH2AX, a phosphorylated histone associated with DNA damage sites. Phosphorylated ATM (pATM^S1981^), an activated upstream kinase in the DDR, also colocalized with GFP-RPA32 at VRCs. These data confirmed that GFP-RPA32 colocalized with VRCs in MuPyV-infected cells.

### 3.3. GFP-RPA32 Relocalizes to VRCs Over Time in MuPyV-Infected Cells

**Figure 3:**
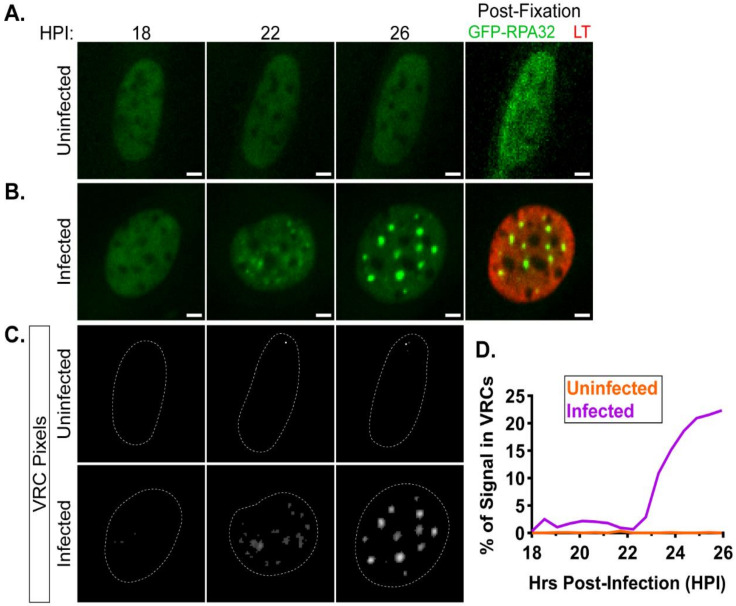
VRC dynamics during live cell microscopy. GFP-RPA32-expressing cells were infected for 18 h and then imaged every 10 min from 18–26 h post-infection (HPI). At 26 HPI, cells were fixed and processed for immunofluorescence microscopy of LT. Representative (**A**) uninfected (LT-negative) and (**B**) infected (LT-positive) cells at 18, 22, and 26 HPI, as well as post-fixation (GFP-RPA32 = green, LT = red). Scale bar = 5 μm. (**C**) Focal pixels were identified for each frame in (**A**,**B**) based on GFP-RPA32 signal. Pixels with intensity values > 1.5× higher than the mean signal intensity of a single nucleus within each frame were defined as “VRC pixels.” The dotted white line in each image denotes the nuclear border (Figure S2A). (**D**) The integrated signal density of VRC-associated pixels was divided by the integrated signal density of the whole nucleus to calculate “% of Signal in VRCs” values. The % of Signal in VRCs values are shown for the uninfected (orange trace) and infected (violet trace) cells shown in (**A**) and (**B**), respectively, between 18–26 HPI.

We next used live cell microscopy of GFP-RPA32 to visualize VRC dynamics during MuPyV infection. Cells were infected (t = 0) and then imaged every 10 min between 18 and 26 h post-infection (HPI). The cells were fixed at the end of the time course, LT was immunolabeled, and the same cells were re-imaged to determine those infected during the time course. Uninfected cells (LT-negative) exhibited small and transient GFP-RPA32 foci, if any (Figure 3A,C, and Video S1). Infected cells (LT-positive) showed GFP-RPA32 foci that colocalized with LT (Figure S2D) and increased in size over the course of infection (Figure 3B,C and Video S2). We term the appearance of foci and their subsequent growth as VRC “formation” and “expansion,” respectively. These data also suggested that the appearance of GFP-RPA32 foci (i.e., VRCs) was sufficient to determine which cells are infected during live cell time course experiments, allowing us to continue collecting images late in infection without requiring the immunolabeling and re-imaging technique described above.

A semi-automated image analysis procedure was developed to quantify GFP-RPA32 localization, as defined by fluorescence intensity. For each nucleus, the nuclear boundary was segmented based on GFP-RPA32 fluorescence (Figure S2A) and a custom MatLab script recorded the fluorescence intensity of each pixel within the nuclear boundary at each time frame (Figure S2B). Kolmogorov–Smirnov nonparametric *t*-tests only indicated significant differences between the three signal distributions in the infected cell (Figure S2B), and three characteristics were observed when comparing the histograms (Figure S2C): (i) the majority of pixels within each nucleus became dimmer over time; (ii) a long, high-intensity tail developed throughout the time course; (iii) the mean nuclear fluorescence intensity did not fluctuate significantly over time. These characteristics suggested that GFP-RPA32 distribution shifted from low-intensity (diffuse) areas to high-intensity (focal) areas throughout the time course, consistent with images of GFP-RPA32 relocalization to VRCs.

Based on the analysis described above, a metric was developed to quantify VRC dynamics over time. For each nucleus at each time point, a floating intensity threshold was calculated and used to identify VRC-associated pixels (Section 2.8.3 and Figure 3C). The integrated GFP-RPA32 signal in those pixels was divided by the total nuclear GFP-RPA32 signal to yield the percentage of VRC-associated signal (Figure 3D). Alternative metrics, such as the percentage of nuclear area occupied by VRCs, exhibited a similar trend but a smaller dynamic range (Figure S2E). Therefore, the percentage of VRC-associated signal could be used to quantify the relocalization of GFP-RPA32 signal from diffuse nuclear areas to focal VRCs.

### 3.4. Kinetics of VRC Formation Depend on Multiplicity of Infection

**Figure 4:**
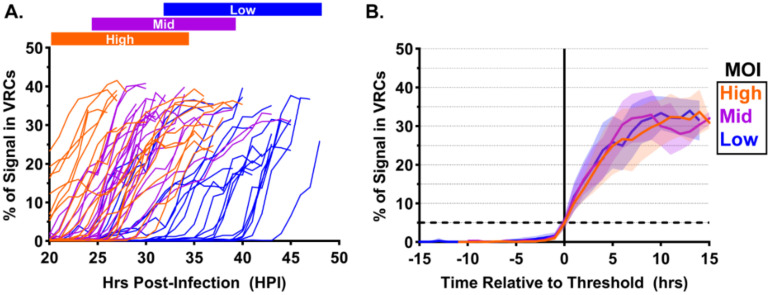
Kinetics of VRC formation depend on multiplicity of infection. GFP-RPA32-expressing cells were infected with MuPyV at one of three multiplicities of infection (MOIs): High (40% infection, orange), Mid (30% infection, violet), or Low (10% infection, blue). Cells were imaged every 12 min from 20–48 HPI and analyzed as described in Figure 3 to yield the proportion of GFP-RPA32 signal associated with VRCs (% of Signal in VRCs) at each time point. (**A**) The “% of Signal in VRCs” shown for individual cells in each condition, plotted as a function of hours post-infection (HPI). Colored boxes above the axes denote the approximate range of VRC formation times for each condition. (**B**) The mean “% of Signal in VRCs” for each condition was calculated after aligning individual cell traces to the frame in which they first exceed 5% of signal in VRCs (horizontal dashed line). Error (Std. Dev.) is denoted by semi-transparent shaded areas. *n* = 20 cells per condition.

Previous observations of cytopathic effects in MuPyV-infected MEFs suggested that infection kinetics may depend on the MOI used for infection, and we therefore hypothesized that VRC formation kinetics may also be affected by MOI. GFP-RPA32-expressing cells were infected with MOIs that yielded approximately 10% (“low”), 30% (“mid”), or 40% (“high”) infection efficiency (Figure S1F). The virus concentration in the high MOI condition was 4- and 16-fold higher than in the mid and low MOI conditions, respectively. Cells were imaged every 12 min from 20–48 HPI and analyzed as described above. We found that MOI was inversely proportional to the timing of VRC formation. For example, some cells infected with the highest MOI exhibited large VRCs at 20 HPI (Figure 4A, orange traces), whereas the middle and low MOIs showed foci by approximately 22–26 HPI (Figure 4A, violet traces) and 28–40 HPI (Figure 4A, blue traces), respectively. VRC formation kinetics for each cell exhibited considerable variability within and between conditions, likely due to the asynchronous nature of the infection [43].

To reduce this variability, we aligned the data points for each cell (the “cell trace”) to a common threshold (Figure 4B), as described in the Methods. Interestingly, aligning individual cell traces significantly reduced the variability of VRC formation kinetics within and between conditions. Furthermore, we did not detect any significant effect of MOI on the maximum percentage of GFP-RPA32 signal associated with VRCs (30–35%), nor the rate of GFP-RPA32 relocalization during VRC expansion. The number of VRCs per nucleus during VRC formation was also similar between MOIs (19–20 ± 6 in each condition). These results suggest that MOI affects the timing of VRC formation but does not affect the kinetics of VRC expansion or the number of VRCs observed during VRC formation.

### 3.5. Small T-Antigen Is Required for VRC Expansion

**Figure 5:**
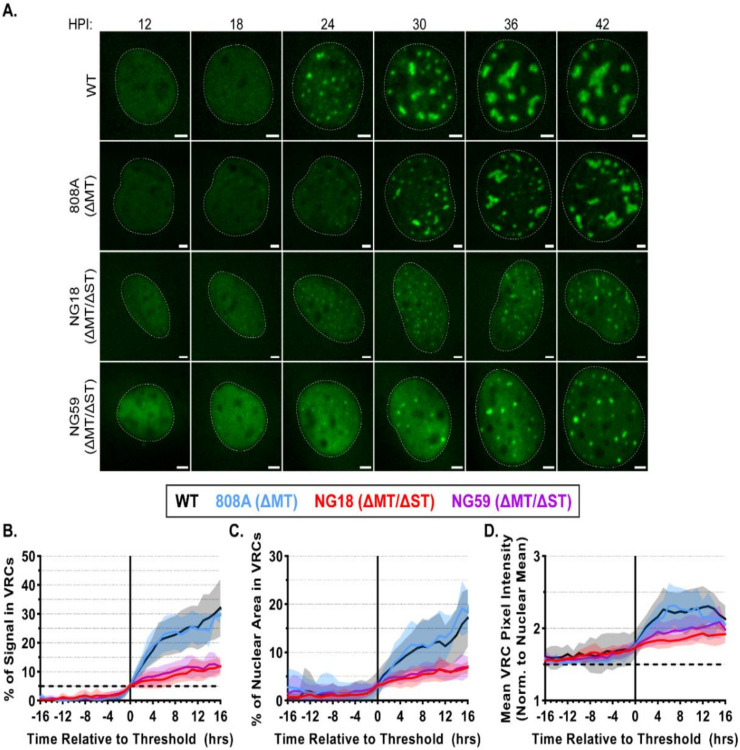
Small T antigen is required for VRC expansion. GFP-RPA32-expressing cells were infected with WT, 808A (ΔMT), NG18 (ΔMT/ΔST), or NG59 (ΔMT/ΔST) MuPyV, imaged every 10 min from 12–48 HPI, and analyzed as described in Figure 3. (**A**) GFP-RPA32 images from the indicated time point (HPI) from a representative cell infected by each virus: WT, 808A, NG18, or NG59. The dotted white lines denote nuclear borders. Scale bar = 5 μm. (**B**–**D**) The mean values of: (**B**) % of Signal in VRCs, (**C**) % of Nuclear Area in VRCs, and (**D**) VRC Pixel Intensity (normalized to nuclear mean intensity) are plotted for cells infected with WT (black), 808A (blue), NG18 (red), or NG59 (violet). Mean values in (**B**–**D**) were calculated after individual traces were aligned, as described in the Methods. The bold dashed line in (**B**) denotes alignment threshold (5% of Signal in VRCs). The bold dashed line in (**D**) denotes VRC pixel threshold (1.5-times the mean nuclear signal intensity). Error (Std. Dev.) is denoted by semi-transparent shaded areas. *n* ≥ 20 cells per condition.

The MuPyV TAg proteins are expressed early in infection and interact with multiple cellular proteins to enhance virus production. Mutant viruses lacking expression of MT and/or ST have been used to separate the activities of these multifunctional proteins. The 808A virus (∆MT) replicates WT levels of vDNA but produces a significantly less infectious virus [21,22]. The NG18 and NG59 viruses (∆MT/∆ST) replicate 10–30% as much vDNA as WT, with a similar reduction in infectious virus production [20,34,35]. Previous studies have also noted the lack of large VRCs in NG18-infected cells [6,7]. To determine how MT or ST affect VRC formation and expansion, we imaged and analyzed GFP-RPA32 relocalization kinetics during WT, 808A, NG18, and NG59 infections. WT and 808A VRCs appeared similar in the time course images, but NG18 and NG59 VRCs did not expand significantly after forming, even immediately prior to cell death (Figure 5A and Videos S3–6). The results of quantitative image analysis were consistent with the above observations, indicating that VRC expansion kinetics were indistinguishable in WT- and 808A-infected cells, and that the percentage of GFP-RPA32 associated with VRCs increased only moderately in NG18- and NG59-infected cells (10–15% on average) (Figure 5B). Interestingly, there was no difference in the number of VRCs observed during VRC formation (19–21 ± 6 per nucleus in each condition).

To investigate further the relocalization of GFP-RPA32 during these infections, we quantified the area and relative signal intensities of VRC pixels over time. Consistent with our observations of the time course images, we found that VRCs in NG18- and NG59-infected cells occupied a smaller proportion of the nuclear area than did VRCs in WT- or 808A-infected cells (5–10% and 15–20%, respectively) (Figure 5C). The intensity of GFP-RPA32 signal in VRC pixels was normalized to the mean nuclear signal and used to estimate the relative density of VRC-associated RPA. We found that, after their formation, NG18 and NG59 VRCs tended to be dimmer (i.e., less dense) than WT and 808A VRCs (1.75–2.25× and 2.00–2.50× brighter than their nuclear means, respectively), suggesting there may be less vDNA-associated RPA at NG18 and NG59 VRCs (Figure 5D). These quantitative analyses are consistent with qualitative observations from fixed cells and suggest that ST, but not MT, is required for VRC expansion, likely reflecting the reduction of vDNA accumulation during NG18 and NG59 infections [6,7,20,35].

## 4. Discussion

We used live cell fluorescence microscopy to study MuPyV VRCs in a mouse fibroblast cell line that stably expresses a GFP-tagged human RPA32 protein. The RPA complex is a known component of VRCs and localizes to both replication- and repair-associated VRC subdomains identified by super-resolution microscopy [7]. Although GFP-RPA32 fusion proteins have been used in previous studies in human cells [44], we validated the appropriate localization of GFP-RPA32 in mouse fibroblasts. These results verified that the GFP-RPA32 protein relocalized to sites of cellular DNA damage (Figure 1 and Figure S1), as well as VRCs in infected cells (Figure 2).

Using GFP-RPA32 to visualize VRCs throughout the course of infection, live cell microscopy captured the dynamic nature of VRCs. In WT-infected cells, we observed small, focal VRCs that expanded over time, often merging into large nuclear tracts that occupied much of the nuclear area. These observations are consistent with fixed cell microscopy images of MuPyV-infected cells, and similar characteristics have been described in other DNA virus replication centers [4,6,45]. A custom image analysis program quantified the progressive relocalization of GFP-RPA32 from the dim nucleoplasm to bright VRCs on a single cell basis (Figure 3 and Figure S2). Although the analysis of individual biomarkers was informative, future studies may use two or more fluorescent biomarkers, imaged simultaneously, to assess the dynamics of processes such as vDNA replication and transcription, the production of monomeric viral genomes associated with the DDR, or viral genome chromatization and encapsidation [6,46]. Image analysis tools that incorporate machine learning or cluster analysis algorithms may also improve the resolution of these studies from the cellular level to that of individual VRCs, and reveal a diversity of composition and organization not presently seen [47,48].

VRC formation began over a range of times post-infection (Figure 4A), likely related to the asynchronicity of the cell cycle and consequent variability in the rates of virus attachment, endocytosis, and genome delivery to the nucleus [49,50,51]. Future studies may improve the analysis by using cell cycle inhibitors to synchronize cells in G_1_ prior to infection [52]. After the VRCs formed, the kinetics of GFP-RPA32 relocalization were relatively consistent between cells and MOI conditions (Figure 4B), suggesting that the rate of vDNA synthesis or the availability of factors required for vDNA synthesis may constrain the VRC expansion rate. Perhaps not surprisingly, we also found that higher MOIs resulted in VRC formation and cell lysis earlier in infection, consistent with a previous study of SV40 in which LT expression and S-phase induction occurred earlier at higher MOIs [43].

The live cell analyses of MuPyV mutant viruses 808A (ΔMT), NG18 (ΔMT/ΔST), and NG59 (ΔMT/ΔST) were consistent with previous results from fixed cells [6,7,53]. Both 808A and WT infections accumulate similar amounts of vDNA [21] and we observed similar kinetics of GFP-RPA32 relocalization between these two viruses (Figure 5, black and blue traces), indicating that MT is not required for vDNA replication or VRC expansion. NG18 and NG59 infections accumulate significantly less vDNA than WT and exhibit smaller VRCs in fixed cells [6,7,20,34,35]. Consistent with the previous results, we saw a reduction in GFP-RPA32 relocalization to VRCs during NG18 and NG59 infections (Figure 5, violet and red), confirming that ST is required for VRC expansion. ST likely enhances vDNA replication through its major binding partner, protein phosphatase 2A (PP2A), by mimicking a B regulatory subunit and thus altering PP2A activity [12,17,54,55,56,57]. The reduced vDNA replication and virus output of NG18 and NG59 infections may result from an inability to efficiently induce and prolong S-phase [16,53,58]. However, the ST proteins of SV40 and Merkel cell PyV are enriched in the nucleus or at VRCs, suggesting that the ST-PP2A complex may directly regulate DNA replication and repair proteins to enhance vDNA replication [59,60,61]. Potentially consistent with both of these models, MuPyV ST is found in both the cytoplasm and the nucleus when it is expressed alone, independently of infection [62,63]. Determining the localization and phosphatase targets of the ST-PP2A complex during MuPyV infection could enhance our understanding of how PyV TAgs mediate infection, as well as how PP2A regulates critical cellular processes.

VRCs may be adjacent to virus assembly factories where encapsidation of viral genomes occurs [5,64]; future studies will investigate this relationship. The spatiotemporal coordination of these sites would enhance infection by recruiting and retaining the required factors nearby. The formation and expansion of VRCs described here may be due to a compartmentalization strategy similar to that previously proposed for herpes simplex virus (HSV) replication, wherein vDNA is more accessible (i.e., less condensed) than cellular DNA, leading to the enrichment of DNA-binding proteins at sites of vDNA replication [65]. For example, MuPyV genome replication requires cellular and viral factors, such as DNA polymerase and LT, that colocalize with viral genomes. These factors become progressively more enriched at those sites as more vDNA is synthesized, and the increased vDNA synthesis creates the expanding VRCs that we observed. The rate of GFP-RPA32 relocalization to VRCs was consistent between cells and could be due to the availability of ssDNA (i.e., the rate of vDNA synthesis), and the failure of NG59 and NG18 VRCs to expand is likely a consequence of decreased vDNA synthesis. Determining how VRCs are formed, maintained, and organized will provide a better understanding of PyV replication, as well as the cellular functions that are co-opted during infection. It is possible that different DNA viruses use similar sets of replication factors during infection, and that the study of their nuclear replication compartments could identify potential targets for antiviral therapeutics.

## Supplementary Materials

The following are available online at https://www.mdpi.com/1999-4915/12/10/1123/s1, Figure S1: Additional characterization of GFP-RPA32- and GFP-NLS-expressing cell lines; Figure S2: Additional analysis of VRC dynamics during live cell microscopy. Video S1: Movie of uninfected GFP-RPA32-expressing cell from Figure 3. Video S2: Movie of infected GFP-RPA32-expressing cell from Figure 3. Video S3: Movie of WT MuPyV-infected GFP-RPA32-expressing cell from Figure 5. Video S4: Movie of 808A-infected GFP-RPA32-expressing cell from Figure 5. Video S5: Movie of NG18-infected GFP-RPA32-expressing cell from Figure 5. Video S6: Movie of NG59-infected GFP-RPA32-expressing cell from Figure 5.
