# Supplementary material for: Live Cell Microscopy of Murine Polyomavirus Subnuclear Replication Centers
Source: Viruses. 2020 Oct 2;12(10):1123. doi: 10.3390/v12101123 (PMC7650712; doi:10.3390/v12101123)
Supplement: Supplementary file 1 [file viruses-12-01123-s001.zip › Live cell RPA supplementarry materials.pdf]

Supplemental Information

Live Cell Microscopy of Murine Polyomavirus Subnuclear Replication Centers

Douglas K. Peters [1], Kimberly D. Erickson [1], and Robert L. Garcea [1,2,*]

[1] BioFrontiers Institute, University of Colorado Boulder, Boulder, Colorado, United States of America
[2] Department of Molecular, Cellular, and Developmental Biology, University of Colorado Boulder, Boulder, Colorado, United States of America
* Correspondence: Robert.Garcea@colorado.edu

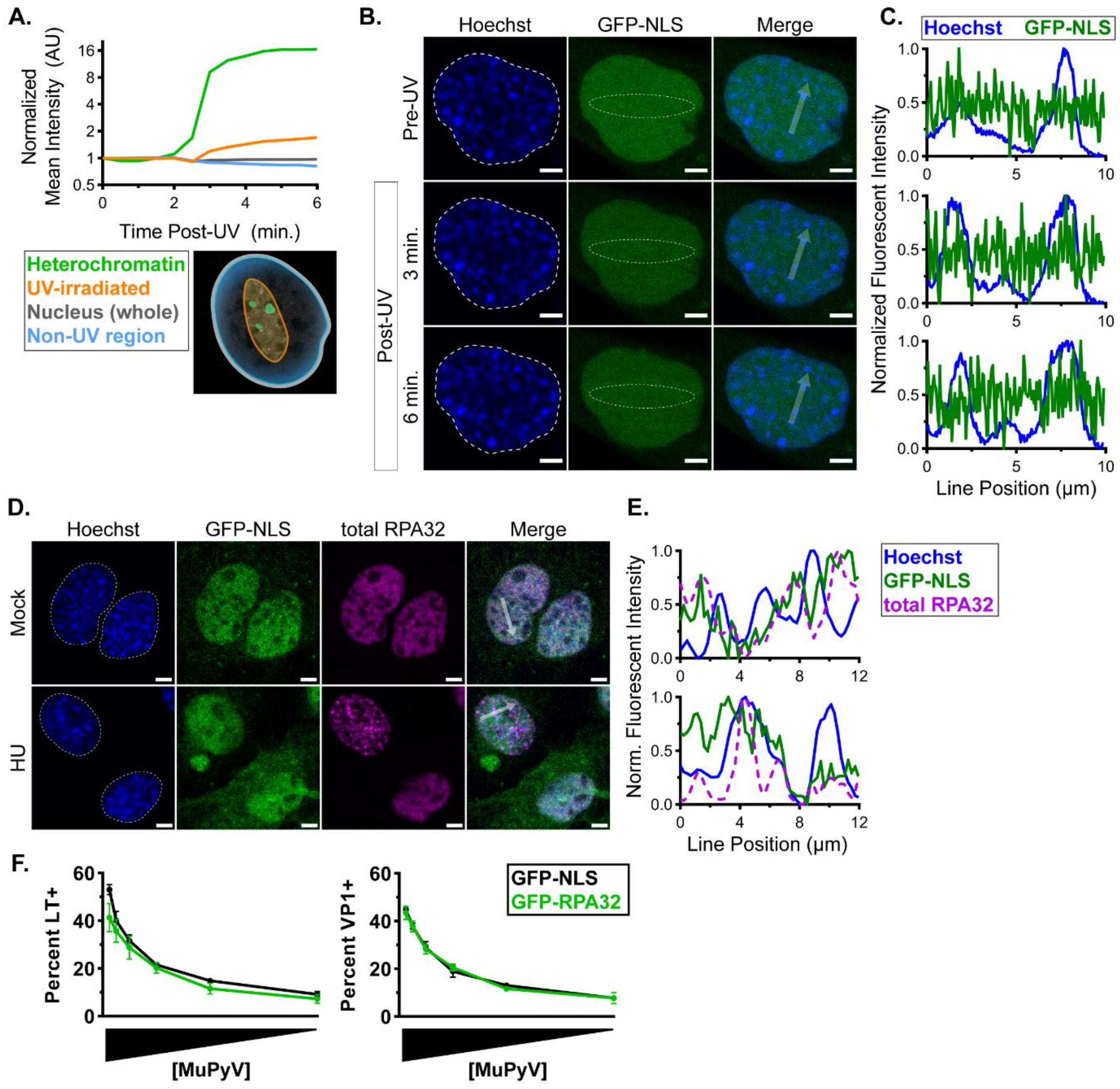

Figure S1: Additional analysis of GFP-RPA32 relocalization to cellular DNA damage. (A) The mean GFP-RPA32 signal intensity was calculated at each time point in each of four nuclear regions: heterochromatin (green), the UV-irradiated zone (orange), the non-irradiated region (blue), and the entire nucleus (gray). Mean intensity values for each nuclear region were normalized to the first time point (pre-UV, t=0 min) to highlight changes in GFP-RPA32 localization over time. **(B)** Mouse cells stably expressing GFP-NLS were imaged before and after induction of cellular DNA damage by UV irradiation as described in Figure 1. Hoechst (blue, left column), GFP-NLS (green, middle column), and merge (right column) images are shown of a representative cell at multiple time points: Pre-UV irradiation (top row), 3 min. post-UV (middle row), and 6 min. post-UV (bottom row). The nuclear area is denoted by a dotted white line in each Hoechst image, and the UV-irradiated region is denoted by a dotted white line in each GFP-NLS image. **(C)** Line scan analysis of Hoechst (blue) and GFP-NLS (green) signals along the white arrow in each merge image in (B). Fluorescence intensities were analyzed for each fluorescent channel and normalized to min and max values within each channel. **(D)** Hoechst (blue, 1st column), GFP-NLS (green, 2nd column), total RPA32 (magenta, 3rd column) and merge (4th column) images are shown in representative cells 1 hr after treatment with HU-free media (Mock, top row) or media containing 5mM HU (bottom row). Nuclear borders are denoted in each Hoechst image by dotted white lines. **(E)** Line scan analysis of Hoechst (blue), GFP-NLS (green), and total RPA32 (magenta) signal along the white arrows in each merge image in (D). Fluorescence intensities were analyzed as in (C). Scale Bars = 5μm. **(F)** GFP-RPA32- and GFP-NLS-expressing cells (green and black lines, respectively) were infected with a range of virus concentrations (left=higher, right=lower), fixed and immunolabeled for LT and VP1, and imaged. The highest concentration of virus is 32-fold higher than the lowest. Automated image analysis determined the average percentage of cells expressing LT (left) or VP1 (bottom) in each condition. Error Bars = Std. Dev. n = 4 replicates per condition.

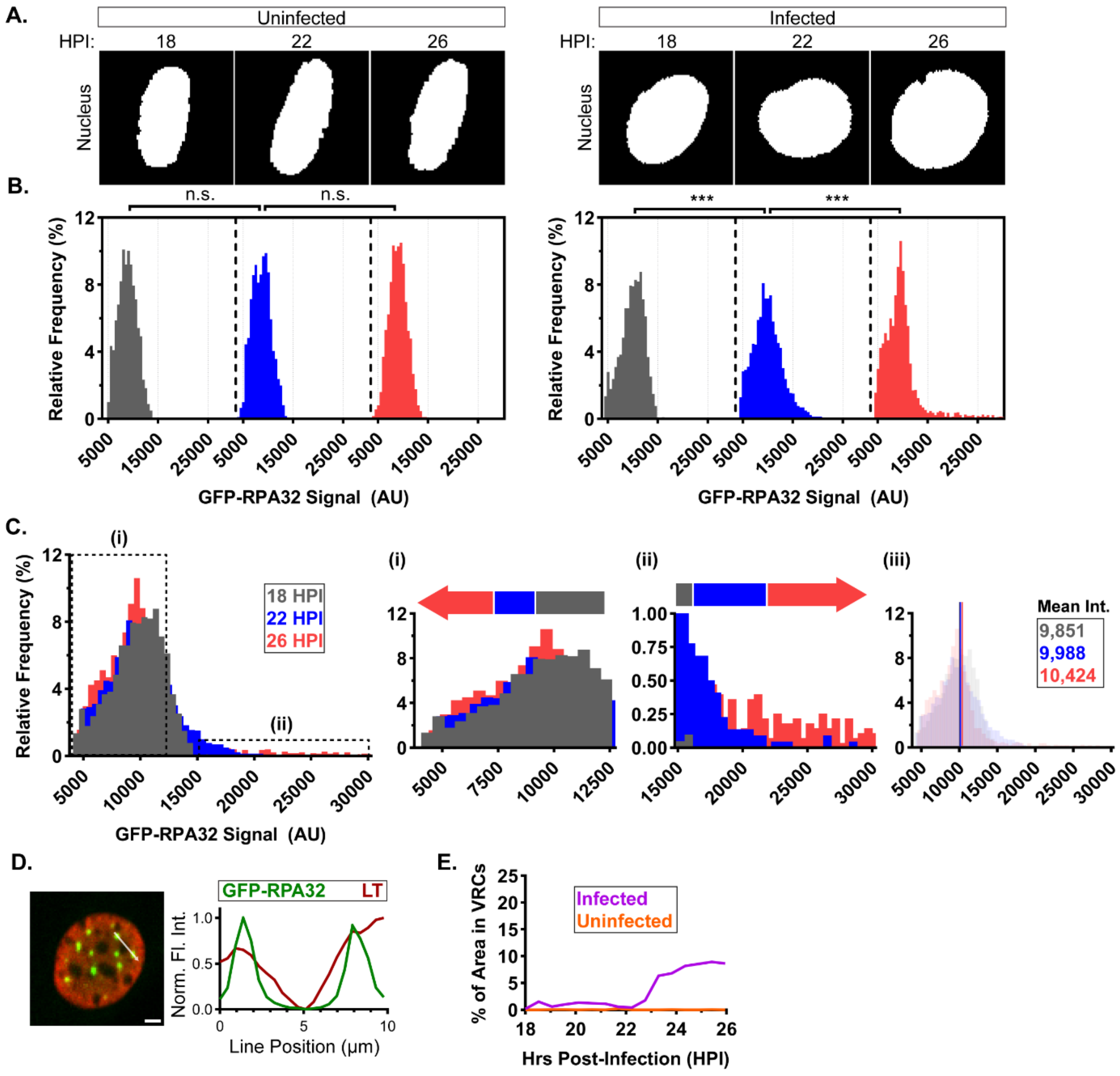

Figure S2: Additional analysis of VRC dynamics during live cell microscopy. (A) Identification of nuclear (white) and non-nuclear (black) pixels for each frame in Figure 3A-B. **(B)** Histograms of nuclear GFP-RPA32 signal distribution for each frame in Figure 3A-B. GFP-RPA32 signal is binned and plotted against the relative frequency (%) of each bin. Kolmogorov-Smirnov nonparametric t-tests were used to compare the cumulative distributions of each data set. n.s. = $p>0.05$; *** = $p<0.001$. **(C)** Histograms from infected cell in (B) shown overlapping for comparison of GFP-RPA32 signal distributions. Dotted boxes (labeled (i) and (ii)) denote cropped regions of the histograms, which are shown to the right with matching labels. (i) The left-shift of the majority of pixels from 18-26 HPI, indicated by arrow above histogram, represents these pixels becoming dimmer over time. (ii) The right-shift of a small proportion of pixels from 18-26 HPI, indicated by arrow above histogram, represents the growing subpopulation of bright VRC pixels. (iii) The mean nuclear GFP-RPA32 signal intensities plotted as vertical lines overlaying signal histograms and reported for each frame, indicating minimal fluctuation despite shifts in GFP-RPA32 signal distribution. **(D)** Post-fixation image of infected cell from Figure 3B and line scan analysis of GFP-RPA32 (green) and LT

(red) signals along the white arow in the image. Fluorescence intensities were analyzed and normalized as in Figure 1. **(E)** The proportion of nuclear area associated with VRCs (% of Area in VRCs) between 18-26 HPI for the uninfected (orange trace) and infected (violet trace) cells shown in (A) and (B), respectively.

Video S1: Movie of uninfected GFP-RPA32-expressing cell (18-26 HPI). Image stack of uninfected cell from Figure 3A. One frame per hour of imaging.

Video S2: Movie of infected GFP-RPA32-expressing cell (18-26 HPI). Image stack of infected cell from Figure 3B. One frame per hour of imaging.

Video S3: Movie of WT MuPyV-infected GFP-RPA32-expressing cell (12-48 HPI). Image stack of WT MuPyV-infected cell from Figure 5. One frame per hour of imaging.

Video S4: Movie of 808A-infected GFP-RPA32-expressing cell (12-48 HPI). Image stack of 808A-infected cell from Figure 5. One frame per hour of imaging.

Video S5: Movie of NG18-infected GFP-RPA32-expressing cell (12-48 HPI). Image stack of NG18-infected cells from Figure 5. One frame per hour of imaging.

Video S6: Movie of NG59-infected GFP-RPA32-expressing cell (12-48 HPI). Image stack of NG59-infected cell from Figure 5. One frame per hour of imaging.
