# Supplementary figures and images for: Live Cell Microscopy of Murine Polyomavirus Subnuclear Replication Centers
Source: Viruses. 2020 Oct 2;12(10):1123. doi: 10.3390/v12101123 (PMC7650712; doi:10.3390/v12101123)

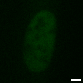

Supplement: Supplementary file 1 [file viruses-12-01123-s001.zip › Video S1 - Uninfected Cell from Figure 3.gif]

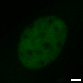

Supplement: Supplementary file 1 [file viruses-12-01123-s001.zip › Video S2 - Infected Cell from Figure 3.gif]

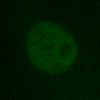

Supplement: Supplementary file 1 [file viruses-12-01123-s001.zip › Video S3 - WT-infected Cell from Figure 5.gif]

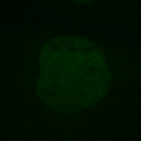

Supplement: Supplementary file 1 [file viruses-12-01123-s001.zip › Video S4 - 808A-infected Cell from Figure 5.gif]

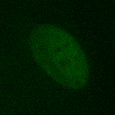

Supplement: Supplementary file 1 [file viruses-12-01123-s001.zip › Video S5 - NG18-infected Cell from Figure 5.gif]

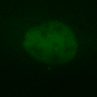

Supplement: Supplementary file 1 [file viruses-12-01123-s001.zip › Video S6 - NG59-infected Cell from Figure 5.gif]
